# Right ventricular assessment of the adolescent footballer’s heart

**DOI:** 10.1186/s44156-023-00039-4

**Published:** 2024-02-29

**Authors:** D. X. Augustine, J. Willis, S. Sivalokanathan, C. Wild, A. Sharma, A. Zaidi, K. Pearce, G. Stuart, M. Papadakis, S. Sharma, A. Malhotra

**Affiliations:** 1 Royal United Hospitals Bath NHS Foundation Trust, Bath, UK; 2 Department for Health, University of Bath, Bath, UK; 3 Division of General Internal Medicine, University of Pennsylvania Health System, Philadelphia, PA USA; 4 Cardiovascular Clinical Academic Group, St George’s, University of London and St George’s University Hospitals NHS Foundation Trust, London, UK; 5 University Hospital of Wales, Cardiff, UK; 6 Institute of Sport, Manchester Metropolitan University and Manchester University NHS Foundation Trust, Manchester, UK; 7 Heart Institute, University of Bristol, Bristol, UK

**Keywords:** Sports cardiology, Cardiomyopathy, Exercise, Athlete, Football, Echocardiography

## Abstract

**Introduction:**

Athletic training can result in electrical and structural changes of the right ventricle that may mimic phenotypical features of arrhythmogenic right ventricular cardiomyopathy (ARVC), such as T-wave inversion and right heart dilatation. An erroneous interpretation may have consequences ranging from false reassurance in an athlete vulnerable to cardiac arrhythmias, to unnecessary sports restriction in a healthy individual. The primary aim of this study was to define normal RV dimension reference ranges for academy adolescent footballers of different ethnicities. Secondary aims include analysis of potential overlap between this adolescent group with ARVC criteria and comparison with normal adult ranges.

**Results:**

Electrocardiographic (ECG) and echocardiographic data of 1087 academy male footballers aged between 13 and 18 years old (mean age 16.0 ± 0.5 years), attending mandatory cardiac screening were analysed. Ethnicity was categorised as white (n = 826), black (African/Caribbean; n = 166) and mixed-race (one parent white and one parent black; n = 95). Arrhythmogenic right ventricular cardiomyopathy major criteria for T-wave inversion was seen in 3.3% of the cohort. This was more prevalent in black footballers (12%) when compared to mixed race footballers (6.3%) or white footballers (1%), P < 0.05. Up to 59% of the cohort exceeded adult reference ranges for some of the right ventricular parameters, although values were similar to those seen in adult footballers. There were no differences in right ventricular dimensions between ethnicities. In particular, the right ventricular outflow tract diameter would fulfil major criteria for ARVC dimension in 12% of footballers. Overall, 0.2% of the cohort would fulfil diagnosis for ‘definite’ arrhythmogenic right ventricular cardiomyopathy and 2.2% would fulfil diagnosis for ‘borderline’ arrhythmogenic right ventricular cardiomyopathy for RV dimensions and ECG changes. This was seen more frequently in black footballers (9.9%) than mixed race footballers (3.9%) or white footballer (0.6%), P < 0.05. Among athletes meeting definite or borderline arrhythmogenic right ventricular cardiomyopathy criteria, no cardiomyopathy was identified after comprehensive clinical assessment, including with cardiac magnetic resonance imaging, exercise testing, ambulatory electrocardiograms and familial evaluation.

**Conclusion:**

Right heart sizes in excess of accepted adult ranges occurred in as many as one in two adolescent footballers. Structural adaptations in conjunction with anterior T-wave inversion may raise concern for ARVC, highlighting the need for evaluation in expert settings.

## Introduction

Cardiovascular adaptation to exercise can occur in individuals who undertake high volumes of aerobic sport. Such cardiac changes seen in athletes termed the ‘athlete’s heart’ (AH), may mimic mild phenotypes of cardiomyopathies. These physiological changes can often be observed on a resting electrocardiogram (ECG) and echocardiogram and can be influenced by demographics including sex, age, ethnicity and sporting discipline [1–3]. It is therefore important to integrate all these factors when assessing athletes and guide investigations appropriately, in order to help distinguish physiological change from a pathological process.

Historically, the left ventricle (LV) in athletes has been characterized in detail with studies demonstrating how physiological adaptation to exercise may be influenced in adolescents and adults by ethnicity [3–5] sporting discipline and gender [1, 6–8]. By contrast, there are fewer studies detailing physiological adaptation to exercise in the right ventricle (RV) [9–11]. Furthermore, the majority of these studies detail the impact of physiological changes in adults as opposed to characterizing right ventricular geometric changes of the adolescent athlete (aged 13–18 years). There are even fewer studies discussing the potential impact of ethnicity on right ventricular adaptation to exercise.

Arrhythmogenic right ventricular cardiomyopathy (ARVC) has an estimated prevalence of between 1 in 1000 and 1 in 5000 [12]. It is characterized by right ventricular pathology and arrhythmias where there is impaired desmosome function. When subjected to mechanical stress this causes myocyte detachment and cell death. Macroscopically, there is fibrous or fibro-fatty replacement of the myocardium affecting the RV. Phenotypic changes of ARVC can manifest on the echocardiogram as increased right ventricular size and as abnormal T wave inversion (TWI) on the electrocardiogram (ECG). These pathological findings can overlap with physiological changes seen in athletes where chamber dilatation and TWI are well recognised [13–17].

Exercise may exacerbate pathology in those with known ARVC or induce phenotypical change in genotype positive carriers at an earlier stage than would have occurred in a sedentary individual. Multicentre post-mortem series (n = 42) in ARVC has shown that the majority of patients with ARVC die suddenly (81%) with nearly half of these occur during exercise [18]. It is therefore critical that sports participants with potential ARVC are advised appropriately.

Given the potential association of exercise with greater adverse effects, guidelines have recommended restriction in competitive sports for those with ARVD [19]**.**

This study aims to characterize right heart size / function and electrical changes in the adolescent heart from a cohort of male academy footballers (aged 13–18). The impact of ethnicity, indexing to body surface area (BSA) is assessed as well as the potential overlap with ARVC criteria.

## Methods

### Study design

A total of 1087 academy male football players within the Football Association (FA) underwent mandatory cardiac screening. All players were aged between 13 and 18 years old (mean age 16.0 ± 0.5 years). Written informed consent for screening was obtained from each player by the team doctor. Written informed consent from a parent or guardian was required for athletes younger than 16 years of age, in accordance with the FA governance department. Athletes underwent assessment with a health questionnaire, physical examination, 12-lead ECG and echocardiography.

For age group comparison of the RV, similar data was collected on footballers > 18 years (n = 114, mean age 21.4 ± 3.0 years, all male).

### Echocardiographic examination

Two-dimensional echocardiography was performed with Philips (CPX50; iE33, Sonos 7500) and GE Vivid I (Tiral, Israel) machines. Right heart size and function measurements were in accordance with national guidelines [20, 21], see Table 1.

**Table 1:** Echocardiographic measurements acquired. Adapted from [20, 21]

Measurements	Explanatory note	Image
Right ventricular outflow tract parasternal long axis view (RVOTP)	Proximal region of the RVOT in PLAX view. Interventricular septum to anterior RV free wall measured in end diastole	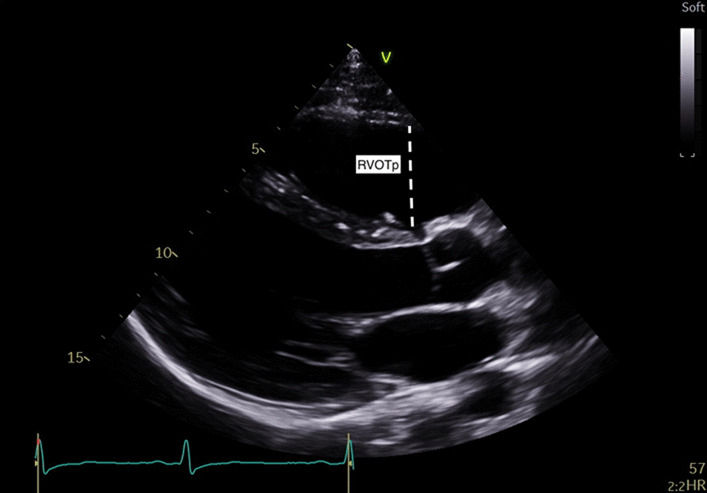
Right ventricular outflow tract short axis view	RVOT-1: Perpendicular to the central point of aortic valve closure line to the endocardial border measured in end diastole RVOT-2: Measurement made just below the pulmonary valve annulus, inner border to inner border measured in end diastole	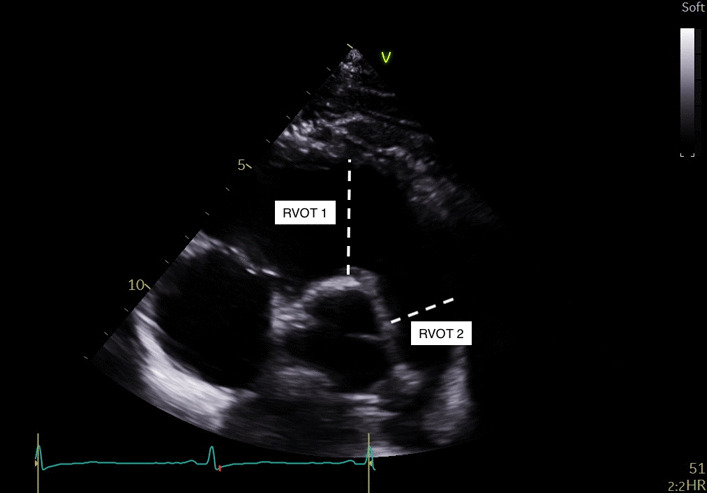
RV Dimensions (RVD1, RVD2, RVD3)	All measurements taken at end diastole RVD1: Basal RV diameter. Measured at the maximal transverse diameter in the basal one third of the RV RVD2: Mid RV diameter measured at the level of the LV papillary muscles RVD3: RV length (from the plane of the tricuspid annulus to the RV apex)	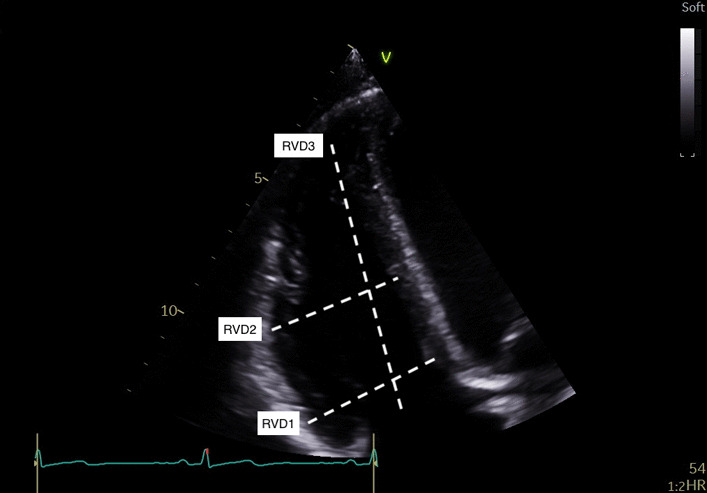
Right atrial area	Measure at end ventricular systole on the frame just prior to tricuspid valve opening. Trace the RA from the plane of the TV annulus along the IAS, superior and lateral walls of RA	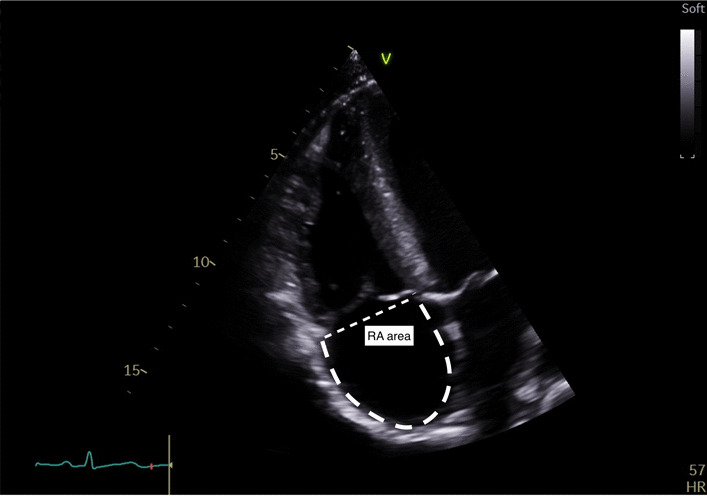
Fractional Area Change (FAC)	Manual tracing of the RV endocardial border from the lateral tricuspid annulus along the free wall to the apex and back along the interventricular septum to medial tricuspid valve annulus at end diastole and end systole FAC = (RVAd−RVAs)/RVAd	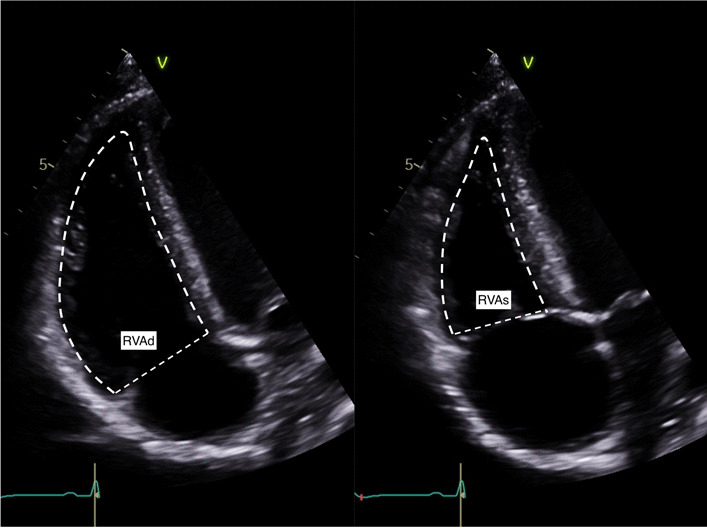
RV pulsed tissue Doppler S Wave (S’) velocity	PW tissue Doppler S wave measurement taken at the lateral tricuspid annulus in diastole. It is important to ensure the basal RV free wall segment and the lateral tricuspid annulus are aligned with the Doppler cursor to avoid velocity underestimation	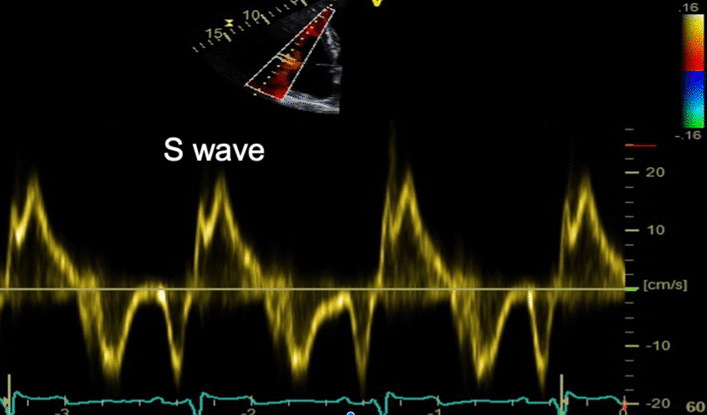
Tricuspid systolic annular plane excursion (TAPSE)	Align the M-Mode cursor along the direction of the lateral tricuspid annulus. Select a fast sweep speed Measure total excursion of the tricuspid annulus	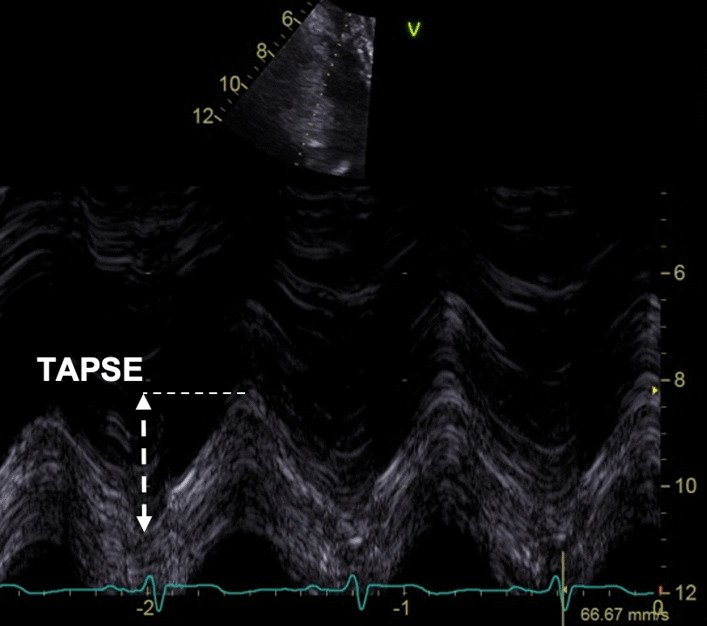

### Statistical analyses

Values are expressed as mean ± standard deviation (SD) or percentages as appropriate. Group differences were analysed using ANOVA (with Tukey post hoc test) or the Kruskal–Wallis test with (with Dunn’s post hoc test) where appropriate. Upper reference values for RV dimensions were calculated as mean + 1.96SD for normally distributed data. For non-normally distributed data reference intervals were calculated (MedCalc Software, Ostend, Belgium) using the 2.5th percentile and 97.5 percentile for the lower and upper intervals respectively. The chi-square test was used to assess proportional differences between groups where appropriate. A 2-tailed probability value < 0.05 was considered to indicate significance. Statistical analysis was performed with SPSS software, version 25 (Chicago, IL).

## Results

### Subjects

Of the total group (n = 1087), mean age was 16.4 ± 0.5y. Eight hundred and twenty six (76%) were white footballers (WFs), 166 (15%) were black footballers (BFs) and 95 (9%) were mixed race footballers (mixed black/white—MFs). BFs had significantly higher weight and BSA than either WFs or MFs (Table 2).

**Table 2:** Background demographics of academy footballers < 18 years

	Black footballers		White footballers		Mixed race footballers		Total		*P*
	Mean ± SD	N	Mean ± SD	N	Mean ± Std. Deviation	N	Mean ± SD	N	
Age (year)	16.4 ± 0.6	166	16.4 ± 0.5	826	16.5 ± 0.6	95	16.4 ± 0.5	1087	0.132
Height (cm)	177.3 ± 6.9	143	178.2 ± 7.0	770	178.1 ± 6.8	92	178.1 ± 7.0	1005	0.327
Weight (Kg)	72.5 ± 9.0	139	69.3 ± 7.8	768	69.6 ± 9.2	89	69.8 ± 8.2	996	< 0.0001*
BSA (m^2^)	1.9 ± 0.1	139	1.9 ± 0.1	762	1.9 ± 0.1	88	1.9 ± 0.1	989	0.049^#^
Training (H/week)	11.2 ± 4.6	98	13.6 ± 5.3	546	14.0 ± 5.7	63	13.3 ± 5.3	707	< 0.0001^#^

*Y* Years, *SD* standard deviation *N* number *CM* centimetres *Kg* kilograms *H* hours

^*^Statistically significant between black footballers and white & mixed race footballers

^#^Statistically significant between black footballers and white & mixed Race footballers

The adult footballers cohort (n = 114) had a mean age of 21.4 ± 3.0y (Table 3). Their mean height, weight and BSA were significantly greater than the academy footballer cohort. Training time (hours per week) was similar between both academy and adult groups (13.3 ± 5.3 vs. 13.9 ± 5.1 h). Academy footballers on average had been training for 8 years. No academy footballer reported any cardiac symptoms suggestive of underlying cardiac pathology or any family history of cardiomyopathy or sudden cardiac death. Self-reported medical conditions included hay fever (n = 14); acne (n = 14); asthma (n = 18); diabetes (n = 1) and juvenile idiopathic arthritis and inflammatory bowel disease (n = 1). One of the cohort was prescribed oral steroids for an underlying medical condition.

**Table 3:** Background demographics comparing academy footballers (age < 18 years) with footballers > 18 years

	Academy footballers < 18 years		Footballers > 18 years		*P*
	Mean ± SD	N	Mean ± SD	N	
Age (y)	16.4 ± 0.5	1087	21.4 ± 3.0	114	0.02*
Height (cm)	178.1 ± 7.0	1005	180.1 ± 7.7	96	0.009*
Weight (Kg)	69.8 ± 8.2	996	77.4 ± 9.8	97	0.0001*
BSA (m^2^)	1.9 ± 0.1	989	2.0 ± 0.2	96	0.001*
Training (H/week)	13.3 ± 5.3	707	13.9 ± 5.1	67	0.294

*Y* Years, *SD* standard deviation, *N* number, *CM* centimetres, *Kg* kilograms, *H* hours

^*^Significant difference between under and over 18s

### RV dimensions in academy footballers—normal ranges

Normal ranges for the various right heart echocardiographic dimensions measured in this study are demonstrated in Table 4 and compared with published normal ranges in adults [22–25] and athletes [10, 23]. For absolute RV dimensions, parameters in the academy footballer cohort exceeded those published in joint American and European guidance [22] by between 5% (for RVD1) to 59% (for RVOT2). Of the 4 adult RV published reference ranges described in Table 4, the ranges for RV dimensions obtained in the current study were most similar to the adult RV normal ranges published by Kou et al. [23] and Addetia et al. [25]. < 10% of any of the measurements were greater than the ranges published by Kou et al. [23] and < 5% were greater than those published by Addetia et al. [25]. Reference ranges from Popple et al. [10] who studied a smaller group of academy footballers (n = 100) were mainly larger than those seen in the current study. The upper limits of reference ranges in the current study (aside to RVOT2) were similar to those published by D’Ascenzi et al. [11] who studied adult competitive athletes from different sporting disciplines.

**Table 4:** Right heart normal ranges from current study compared with published ranges in non athlete adults and adolescent/adult athletes

Normal reference ranges	Current study	Adult normal Lang et al. [22]	Adult normal Willis et al. [24]	Adult Normal Kou et al. [23]	Academy footballers Popple et al. (mean age 16 years) [10]	Adult Athletes D’Ascenzi et al. [11]	% Current study > published normal adult reference ranges				% Current study > published RV athlete reference ranges	
	Academy Footballers (mean age 16 years)						Lang et al. [22]	Willis et al. [24]	Kou et al. [23]	WASE [25]	Popple et al. [10]	D’Ascenzi et al. [11]
RVOTp (mm)	19–35	20–30	19–33	25–43	22–38	26–33	22	3	0	0.1	0.1	3
RVOT1 (mm)	20–37	21–35	21–37	24–44	20–40	32–35	6	2	0.1	1	0.5	6
RVOT2 (mm)	17–30	17–22	16–25	16–29	18–30	15–18	59	23	3	1	3	90
RVD1 (mm)	28–45	25–41	24–38	26–47	32–48	38–42	5	27	0.2	1	0.1	7
RVD2 (mm)	21–39	19–35	22–39	19–42	20–40	27–39	9	9	1	1	1	1
RVD3 (mm)	60–92	59–83	68–88	55–87	66–98		16	4	6	1	0.3	
Indexed RVOTp (mm/m^2^)	10–19		10–18		18–30	15–18		3			0	3
Indexed RVOT1 (mm/m^2^)	11–20		12–21		16–34	16–20		1			0	2
Indexed RVOT2 (mm/m^2^)	9–16		8–15		14–26	15–18		5			0	0
Indexed RVD1 (mm/m^2^)	15–25		13–21		25–37	19–26		21			0	1
Indexed RVD2 (mm/m^2^)	11 – 21		11–23		20–40	14–22		1			0	1
Indexed RVD3 (mm/m^2^)	31–50		34–48		53–73			5			0	

*RVOTP* right ventricular outflow tract parasternal long axis view, *RVOT1/2* right ventricular outflow tract proximal/distal diameter, *RVD1/2/3* RV diameter basal/mid/base to apex; *mm* millimetres, *Y* years

### Electrocardiographic characteristics

TWI in precordial leads was observed in leads V1-V2, V1-V3 or V1-V4 in 4.3%, 3.3% and 1.3% of the cohort, respectively. These patterns of TWI were more frequently seen in BFs than MFs or WFs. TWI in leads V1-V2 was seen in 13% of BF compared with 7.3% of MFs and 2.2% of WFs (P = 0.005). TWI in leads V1-V3 was seen in 13% of BFs compared with 6.3% of MFs and 1% of WFs (P = 0.005). TWI in leads V1-V4 was seen in 4.2% of BFs compared with 2.1% of MFs and 0.6% of WFs (P = 0.001). TWI inferiorly or laterally was a rare occurrence, being seen in 0.1% and 0.2% of the cohort respectively. ECG characteristics of the whole academy footballer group and comparisons between ethnicity is shown in Table 5.

**Table 5:** Comparison of ECG data in academy footballers according to ethnicity

	BF	WF	MF	Whole group	P
Heart rate bpm (n)	59 ± 10 (162)	61 ± 11 (791)	61 ± 10 (95)	61 ± 11 (1048)	0.014*
pRBBB, % (n)	1.2 (166)	5.6 (826)	3.1 (95)	4.7 (1087)	0.04***
RBBB, % (n)	0 (166)	0.5 (826)	2.1 (95)	0.6 (1087)	0.08
LBBB, % (n)	0 (166)	0 (826)	0 (95)	0 (1087)	–
LVH, % (n)	45 (166)	28 (826)	36 (95)	31 (1087)	0.004**
RVH, % (n)	0.6 (166)	3.1 (826)	6.3 (95)	3 (1087)	0.03^
Twi V1–V2, % (n)	13 (166)	2.2 (826)	7.3 (95)	4.3 (1087)	0.005**
Twi V1–V3% (n)	13 (166)	1 (826)	6.3 (95)	3.3 (1087)	0.005**
Twi V1–V4, % (n)	4.2 (166)	0.6 (826)	2.1 (95)	1.3 (1087)	0.001**
Twi Inferiorly, % (n)	0.6 (166)	0 (826)	0 (95)	0.1 (1087)	0.06
Twi laterally, % (n)	0.6 (166)	0.1 (826)	0 (95)	0.2 (1087)	0.38
RAE % (n)	1.2 (164)	0.1 (825)	0 (95)	0.3 (1087)	0.05
LAE % (n)	2.4 (166)	2.2 (826)	1 (95)	2.1 (1087)	0.74
QRS duration % (n)	97 ± 13 (164)	100 ± 24 (809)	97 ± 31 (95)	100 ± 22 (1068)	0.23
QT % (n)	399 ± 30 (148)	398 ± 31 (809)	403 ± 30 (87)	398 ± 30 (1044)	0.26
QTc % (n)	390 ± 23 (163)	398 ± 25 (801)	396 ± 25 (93)	397 ± 25 (1057)	0.06
PR % (n)	159 ± 34 (164)	151 ± 51 (800)	158 ± 31 (92)	153 ± 47 (1056)	0.26
LAD % (n)	0 (164)	1.6 (796)	0(90)	1.1 (1050)	0.3
RAD % (n)	0 (164)	0.4 (796)	0 (90)	0 (1050)	-
Normal axis % (n)	100 (164)	98.3 (796)	100 (90)	98.9 (1050)	0.14

*Bpm* beats per minute, *pRBBB* partial right bundle branch block, *RBBB* right bundle branch block, *LBBB* left bundle branch block, *LVH* left ventricular hypertrophy, *RVH* right ventricular hypertrophy, *Twi* T wave inversion, *RAE* right atrial enlargement, *LAE* left atrial enlargement, *LAD* left axis deviation, *RAD* right axis deviation, *QT* QT interval, *QTc* corrected QT

^*^Statistically significant between black footballers and white footballers

**Statistically significant between black footballers and white and mixed race footballers

***Statistically significant between white footballers and black and mixed race footballers

^Statistically significant between mixed footballers and black and white footballers

### Comparison with ARVC diagnostic criteria

**Fig. 1:**
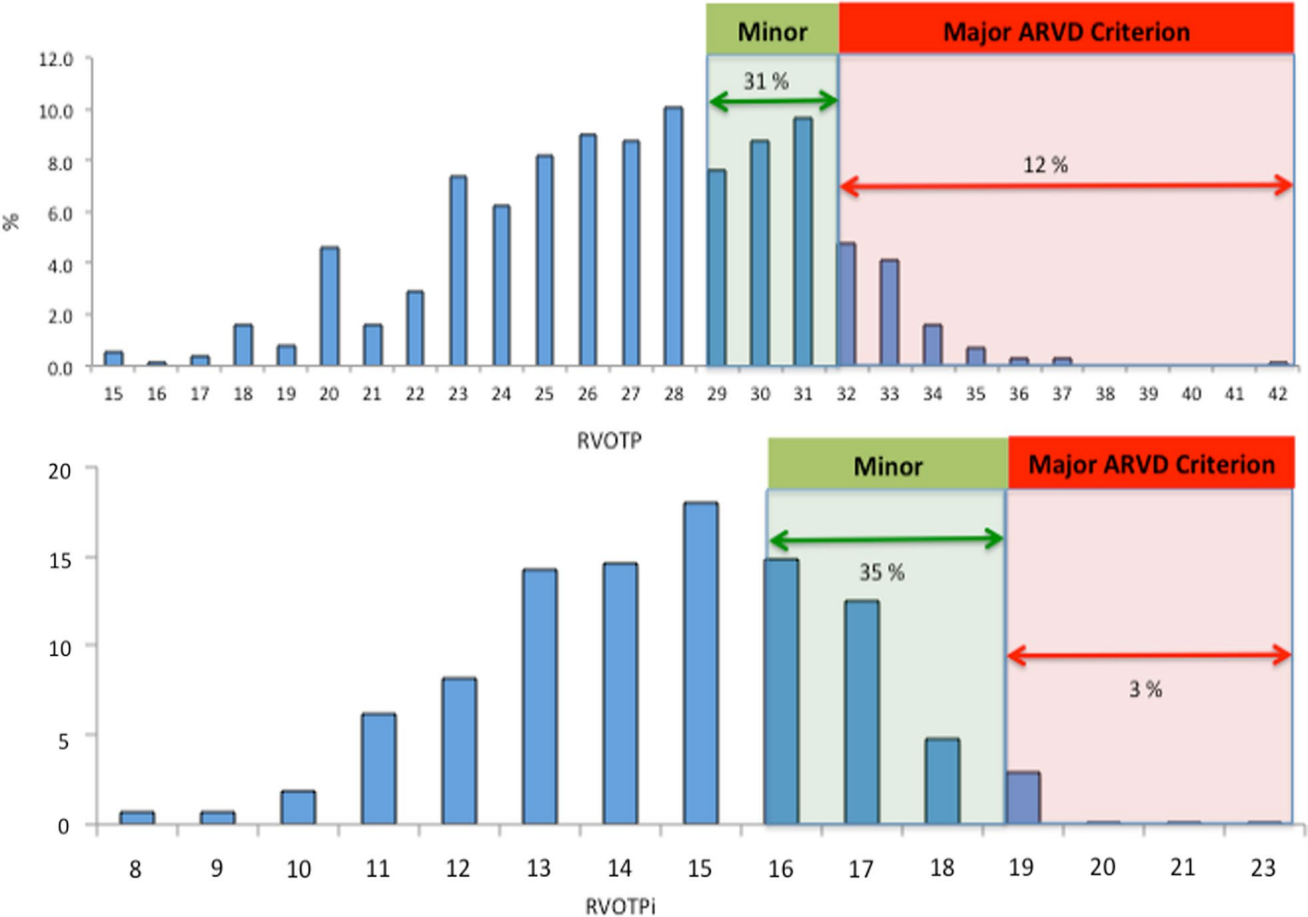
Distribution of values for parasternal long axis right ventricular outflow tract dimensions in adolescent footballers. Absolute dimensions are shown in upper chart and indexed values in lower chart

**Fig. 2:**
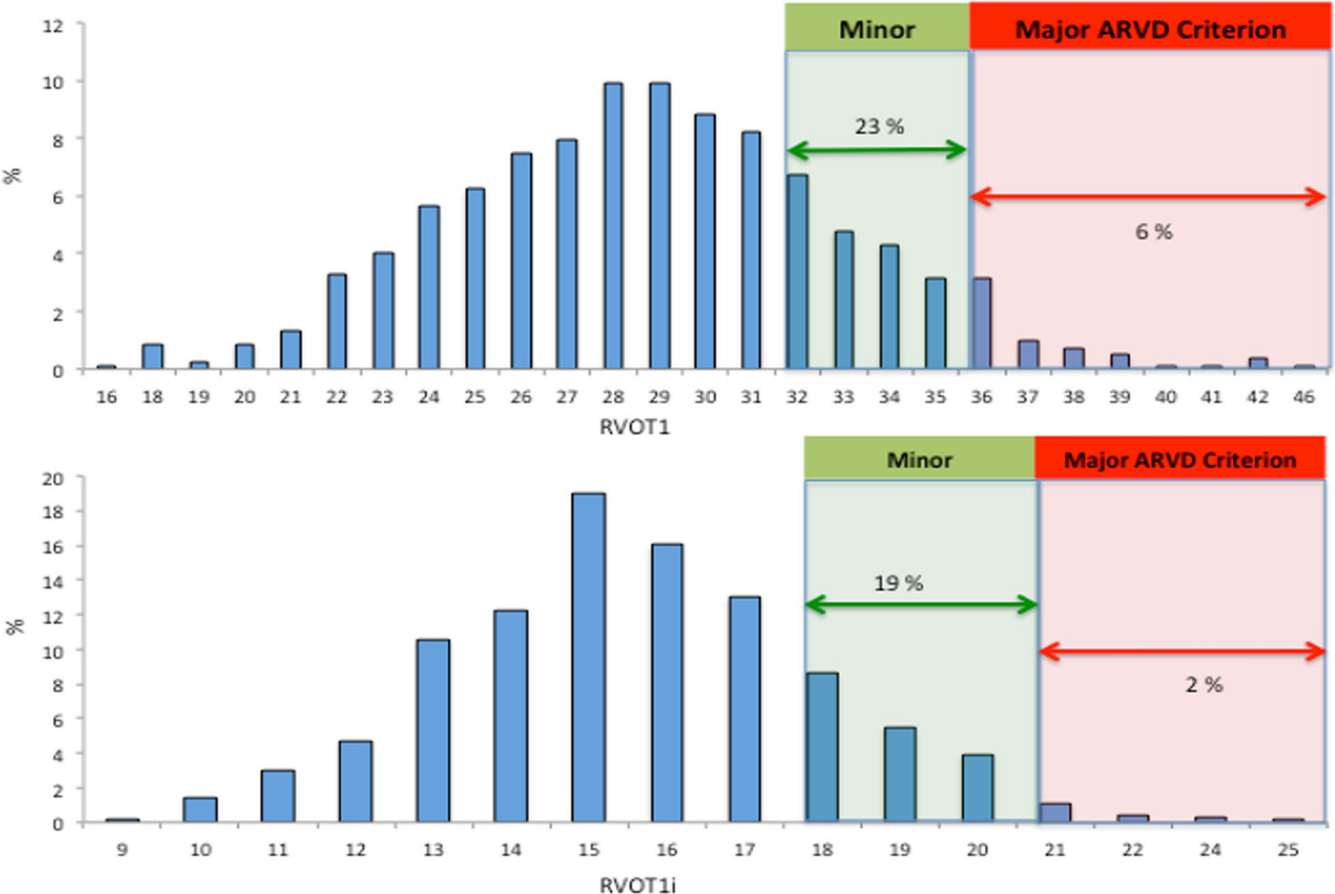
Distribution of values for parasternal short axis right ventricular outflow tract dimensions (RVOT1) in adolescent footballers. Absolute dimensions are shown in upper chart and indexed values in lower chart

There was significant overlap with certain ARVC parameters for academy football players (Table 6). 12% of the academy football cohort would fulfil major absolute dimension ARVC criteria for RVOTp which fell to 3% following indexing for BSA (Fig. 1). Similarly, 6.1% of footballers would fulfil major criteria for absolute RVOT1 dimension and 23% would fulfil minor criteria. When indexed, these values fell to 2% and 19%, respectively (Fig. 2). 0.4% of the cohort fulfilled ARVC major criteria for RV FAC and 12.1% reached thresholds for minor criteria. Overlap between ECG changes observed in the cohort of academy footballers and criteria for ARVC was also seen. ARVC major criteria for TWI was seen in 3.3% of the cohort. This was more prevalent in BFs (12%) when compared to MFs (6.3%) or WFs (3.3%), P = 0.005. Overall, with the physiological changes in RV dimensions and ECGs seen, 0.2% of the cohort would fulfil diagnosis for ‘definite’ ARVC (for dimension and ECG changes). 2.2% of the cohort would fulfil diagnosis for ‘borderline’ ARVC for dimension and ECG changes and this was seen more frequently in BAs (9.9%) compared with MAs (3.9%) or WAs (0.6%), P = 0.0005. Following further evaluation with cardiac MRI, exercise testing, signal averaged ECG, holter monitoring and familial evaluation, no footballer was diagnosed with ARVC.

**Table 6:** Comparison of ECG and echocardiographic data from academy footballers against criteria for ARVC

	BF	WF	MF	Whole group	P
ARVC definite criteria % (n)	0.9 (111)	0.2 (630)	0 (76)	0.2 (817)	0.31
ARVC borderline criteria % (n)	9.9 (111)	0.6 (630)	3.9 (76)	2.2 (817)	0.0005*
RVOTp major % (n)	6.1 (99)	13.2 (570)	9.1 (66)	11.8 (735)	0.1
RVOTp minor % (n)	35 (99)	30 (570)	29 (66)	31 (735)	0.55
RVOT1 major % (n)	6.3 (111)	5.9 (630)	7.8 (76)	6.1 (817)	0.78
RVOT1 minor % (n)	22 (111)	21.9 (630)	23.6 (76)	23 (817)	0.19
RVFAC major % (n)	0 (88)	0.5 (425)	0	0.35 (564)	0.72
RVFAC minor % (n)	13.6 (88)	12.5 (425)	5.6 (51)	12 (564)	0.35
ECG Twi major % (n)	12 (166)	1 (826)	6.3 (95)	3.3 (1087)	0.005*
ECG Twi minor % (n)	1.2 (166)	1.1 (826)	2.1 (95)	1.2 (1087)	0.1

*ARVD* arrhythmogenic right ventricular dysplasia, *RVOTP* right ventricular outflow tract parasternal long axis view, *RVOT1* right ventricular outflow tract proximal, *RV FAC* right ventricle fractional area change, *ECG* electrocardiogram, *BF* black footballer, *WF* white footballer, *MF* mixed race footballer, *N* number, *TWI* T wave inversion

^*^Statistically significant between Black footballers and White & Mixed Race footballers

### RV functional and geometric parameters in academy and adult footballers

Right heart size (absolute and indexed dimensions) and functional echocardiographic results for academy and adult footballers are reported in Table 7. TAPSE was significantly higher in adults compared with academy players (24.5 ± 4.6 mm vs. 24.0 ± 3.7 mm). For both groups, mean values for functional parameters (RV FAC; S’ RV and TAPSE) were within normal published limits and there was no significant difference between age groups for S’ RV and RV FAC. Right atrial dimensions were similar between the groups.

**Table 7:** Echocardiographic RV data for academy and adult footballers

	Academy footballers		Footballers > 18 years		Total		*P*
	Mean ± SD	N	Mean ± SD	N	Mean ± SD	N	
TAPSE (mm)	24.0 ± 3.7	1027	25.4 ± 4.6	100	24.1 ± 3.8	1127	0.008*
RAA (cm^2^)	15.9 ± 2.7	441	16.3 ± 3.5	51	15.9 ± 2.8	492	0.658
RVOTP (mm)	27.0 ± 4.0	735	27.6 ± 4.2	38	27.0 ± 4.1	773	0.324
RVOT1 (mm)	28.8 ± 4.3	817	30.6 ± 4.1	42	28.9 ± 4.3	859	0.003*
RVOT2 (mm)	23.3 ± 3.1	786	23.8 ± 3.6	42	23.4 ± 3.1	828	0.622
RVD1 (mm)	36.3 ± 4.2	1051	36.1 ± 5.2	97	36.3 ± 4.3	1148	0.862
RVD2 (mm)	30.2 ± 4.4	1044	29.8 ± 5.1	95	30.2 ± 4.5	1139	0.379
RVD3 (mm)	75.9 ± 7.8	1030	76.0 ± 8.8	93	75.9 ± 7.9	1123	0.477
RV FAC (%)	49.6 ± 7.8	564	50.0 ± 9.2	33	49.6 ± 7.8	596	0.791
S’ RV (cm/s)	14.8 ± 2.9	202	14.2 ± 2.2	19	14.7 ± 2.9	221	0.395
INDEXED RAA (cm^2^/m^2^)	8.5 ± 1.4	426	8.1 ± 1.7	46	8.5 ± 1.4	472	0.054
INDEXED RVOTP (mm/m^2^)	14.5 ± 2.2	693	13.9 ± 2.5	32	14.5 ± 2.2	725	0.193
INDEXED RVOT 1 (mm/m^2^)	15.5 ± 2.4	762	15.8 ± 2.3	36	15.5 ± 2.4	798	0.303
INDEXED RVOT 2 (mm/m^2^)	12.6 ± 1.7	733	12.3 ± 2.0	36	12.6 ± 1.7	769	0.234
INDEXED RVD 1 (mm/m^2^)	19.6 ± 2.5	964	18.5 ± 2.9	86	19.5 ± 2.5	1050	0.009*
INDEXED RVD 2 (mm/m^2^)	16.2 ± 2.5	958	15.5 ± 2.9	85	16.1 ± 2.6	1043	0.054
INDEXED RVD 3 (mm/m^2^)	40.7 ± 4.5	944	38.6 ± 4.6	83	40.6 ± 4.5	1027	0.001*

*TAPSE* tricuspid annular plane systolic excursion, *Cm* centimetres, *Mm* millimetres, *RAA* right atrial area, *RVOTP* RIGHT ventricular outflow tract parasternal long axis view, *RVOT1/2* right ventricular outflow tract proximal/distal diameter, *RVD1/2/3* RV diameter basal/mid/base to apex, *RV FAC* right ventricle fractional area change, *S’ RV* RV pulsed tissue Doppler S wave velocity

^*^Significant difference between Under and Over 18 s

Analysis of absolute right ventricular dimensions demonstrated that only the RVOT-1 dimension was significantly higher in the adult footballers when compared to the younger academy players. When indexed to BSA this difference was not apparent. There was no significant difference in absolute measurements between the groups for the remainder of the right ventricular outflow or the right ventricular dimensions. When indexed to BSA the academy players (with significantly smaller BSA when compared to adult footballers) demonstrated larger RVD1 and RVD3 values.

### Impact of ethnicity

Functional and geometric echocardiographic parameters were compared between ethnicities in the academy cohort (Table 8). All mean values for functional parameters (TAPSE; RV FAC and S’ RV) were normal and there were no significant differences between ethnicities. There were no significant differences in either absolute or indexed measurements between ethnicities for any of the right heart dimension measures undertaken.

**Table 8:** Impact of ethnicity on echocardiographic RV data for academy footballers

	Black footballers		White footballers		Mixed race footballers		*P*
	Mean ± SD	N	Mean ± SD	N	Mean ± SD	N	
TAPSE (mm)	24.2 ± 3.4	155	23.9 ± 3.8	786	24.3 ± 3.7	86	0.360
RAA (cm^2^)	16.7 ± 2.8	49	15.8 ± 2.7	349	16.0 ± 2.5	43	0.078
RVOTP (mm)	27.4 ± 3.5	99	26.9 ± 4.2	570	27.0 ± 3.7	66	0.648
RVOT1 (mm)	28.5 ± 4.0	111	28.8 ± 4.3	630	29.1 ± 4.7	76	0.501
RVOT2 (mm)	24.0 ± 3.3	107	23.2 ± 3.1	606	23.3 ± 3.0	73	0.156
RVD1 (mm)	36.8 ± 4.4	157	36.2 ± 4.2	801	36.6 ± 3.8	93	0.253
RVD2 (mm)	30.6 ± 3.9	156	30.2 ± 4.5	796	29.5 ± 4.1	92	0.099
RVD3 (mm)	76.4 ± 7.7	155	75.7 ± 7.8	785	76.6 ± 8.1	90	0.300
RV FAC (%)	49.0 ± 7.0	69	49.6 ± 7.9	438	50.2 ± 8.2	57	0.775
S’ RV (cm/s)	13.5 ± 3.7	21	14.9 ± 2.8	171	16.3 ± 2.2	10	0.071
INDEXED RAA (cm^2^/m^2^)	8.8 ± 1.3	57	8.5 ± 1.4	328	8.6 ± 1.7	41	0.483
INDEXED RVOT P (mm/m^2^)	14.5 ± 2.2	92	14.5 ± 2.2	538	15.0 ± 2.2	63	0.344
INDEXED RVOT1 (mm/m^2^)	15.2 ± 2.2	99	15.6 ± 2.4	593	15.5 ± 2.4	70	0.212
INDEXED RVOT2 (mm/m^2^)	12.7 ± 1.6	95	12.6 ± 1.7	570	12.5 ± 1.7	68	0.633
INDEXED RVD1 (mm/m^2^)	19.6 ± 2.7	135	19.5 ± 2.4	743	19.7 ± 2.6	86	0.844
INDEXED RVD2 (mm/m2)	16.2 ± 2.5	135	16.2 ± 2.6	738	15.9 ± 2.5	85	0.336
INDEXED RVD3 (mm/m^2^)	40.6 ± 4.9	133	40.7 ± 4.4	728	41.3 ± 5.1	83	0.578

*SD* standard deviation, *TAPSE* tricuspid annular plane systolic excursion, *Cm* centimetres, *Mm* millimetres, *RAA* right atrial area, *RVOTP* right ventricular outflow tract parasternal long axis view, *RVOT1/2* right ventricular outflow tract proximal/distal diameter, *RVD1/2/3* RV diameter basal/mid/base to apex, *RV FAC* right ventricle fractional area change, *S’ RV* RV pulsed tissue Doppler S wave velocity

### Discussion

This study is the largest study to date to provide normal RV dimension data reference ranges for academy footballers of different ethnicities. We highlight that physiological adaptation due to sporting activity can cause increased RV size in adolescents. The RV dimensions of this cohort of academy footballers is larger than joint reference ranges for adults published by the American and European society of echocardiography [22]. In the current study, up to 59% of certain measurements exceeded those of normal adult ranges published by Lang et al. [22]. The adult reference ranges produced Kou et al. [23] and Addetia et al. [25] are most comparable to the RV dimension ranges for academy footballers obtained in this study. Absolute RV dimensions in academy footballers were similar to adult footballers.

The finding of increased RV dimensions in this study of academy footballers has not been previously appreciated in this age group of footballers with this sample size. RV dimensions have been shown by Zaidi et al. to be greater in athletes (mean age 22 years) when compared to controls [26]. Similar findings were demonstrated by Baggish et al. [27] in elite rowers (mean age 25 years) when compared with controls.

Comparisons of RV physiological adaptation to exercise has also been made in endurance trained athletes (ET) and resistance trained athletes (RT). Utomi et al. [28] described ET RV adaptation (increased absolute diastolic area) in ET athletes (mean age 34 years) but there was limited structural changes noted in RT athletes (mean age 21 years). The findings of enlarged RV measurements in ET athletes was also identified by D’Andrea et al. [9] who analysed RV structural changes in 650 elite athletes (395 ET, mean age 29 years) and 255 (RT). Here, it was seen that RV diameters as well as right ventricular outflow tract (RVOT) diameters were significantly greater in ET athletes than either RT athletes or controls.

Differentiating RV physiological change from potential ARVC is important in those undertaking exercise due to the influence of sports participation on the progression of ARVC. Given that desmosomes play a pivotal role in intercellular integrity, endurance athletes with the genetic predisposition for ARVC are hypothesized to be most at risk for phenotypic expression. Ruwald et al. assessed the impact of exercise on probands diagnosed with ARVC [29]. Here those who participated in competitive sport had an increased risk of ventricular arrhythmia or death (hazard ratio 2.05) when compared with those who were inactive or undertook recreational sport. Furthermore symptoms developed at an earlier stage in the competitive sporting group (30 years vs. 38–41 years). In support of this, it has been reported that competitive sports activity can increase the risk of sudden cardiac death in young adults by five times [30].

Saberniak et al. assessed the impact of exercise on ARVC patients (n = 65) and their genotype positive family members (n = 45). In both groups, vigorous exercise (defined as ≥ 1440 metabolic equivalents / week over 6 years) was associated with biventricular dysfunction when compared with non athletes [31]. This notion of exercise being a trigger to deleterious sequelae of genetic mutations is supported by animal models [32] and more recently in human studies by James et al. They found increased risk of VT, heart failure and ARVC in those who are desmosomal mutation carriers who undertake endurance exercise and frequent exercise [33].

Zaidi et al. previous demonstrated overlap between physiological adaptation with Task force criteria for ARVC in adult athletes (mean absolute age 22 years) [34]. Here, ‘possible’ or ‘borderline’ criteria were seen in 51.1% and 44.5% of the cohort respectively. Our current study is the first to demonstrate significant overlap between right heart measures and ARVC task force criteria in a large cohort of academy footballers. 0.2% of the whole cohort fulfilled ‘definite’ criteria for ARVC based on RV dimension and ECG changes (compared with published ranges of ARVC incidence of 0.001–0.0002%). ‘Borderline’ criteria for ARVC was present in 2.2% of the cohort with a significant higher incidence in black (9.9%) over mixed race (3.9%) or white footballers (0.6%). These differences are largely driven by ethnic variation of T wave inversion seen on ECG. The task force criteria for the diagnosis of ARVC [35] includes the presence of TWI (V1–V3 or beyond) as potential criteria for the presence of ARVC.

In this study, standard RV function parameters were normal. However, advanced RV function techniques such as RV speckle tracking echocardiography (STE) may be of use. Dorobantu et al. [36] found that in athletes with RVOT dilatation, STE evaluation of the RV can demonstrate normal function and differentiate physiological remodelling from pathological changes in arrhythmogenic cardiomyopathy, potentially improving screening in grey-area cases.

### Study limitations

The cohort size varied between the ethnic groups but was a reflection of a true world sample of academy footballers. This is an all-male cohort and results should not be extrapolated to female athletes. The data obtained were from one time point rather than a longitudinal observational study which would have allowed direct comparison of individual cardiac geometric and ECG changes over time. This study has not presented reproducibility data for echocardiographic measurements although all echocardiograms were performed by British Society of Echocardiography accredited echocardiographers.

### Conclusion

**Fig. 3:**
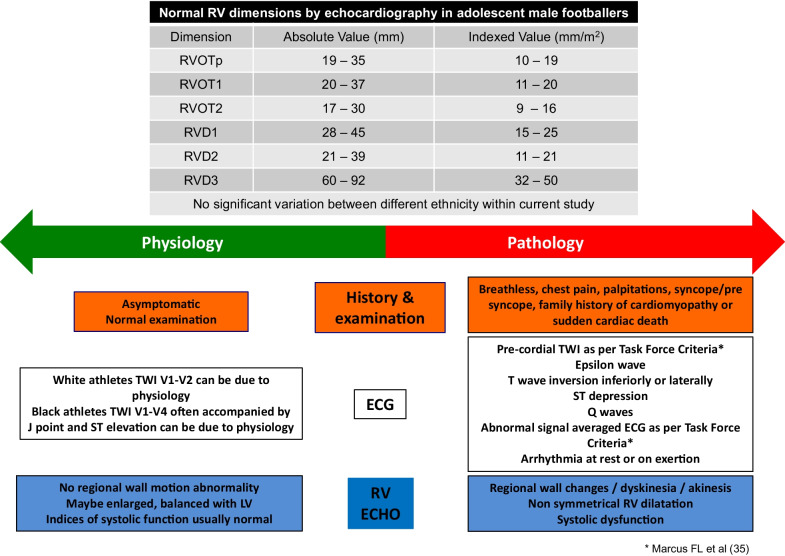
Normal RV dimensions in adolescent male footballers and factors that can help to distinguish physiology from pathology

This study has characterized the male academy footballer’s right heart. Right heart sizes in excess of some standard adult ranges occur frequently in academy footballers and are similar to those seen in adult footballers. It is not unusual to observe values that would overlap with criteria for ARVC. There was no inter-ethnic variability for RV dimensions identified. This work will be of value to those undertaking RV assessment in similar sporting participants (Fig. 3).

## Data Availability

The datasets used and/or analysed during the current study are available from the corresponding author on reasonable request.

## Abbreviations

AH: Athlete’s heart’
ARVC: Arrhythmogenic right ventricular cardiomyopathy
BFs: Black footballers
BPM: Beats per minute
BSA: Body surface area
ECG: Electrocardiogram
FA: Football Association
LBBB: Left bundle branch block
LAD: Left axis deviation
LAE: Left atrial enlargement;
LV: Left ventricle
LVH: Left ventricular hypertrophy
MFs: Mixed race footballers
pRBBB: Partial right bundle branch block
QT: QT interval
QTc: Corrected QT interval
RAA: Right atrial area
RAD: Right axis deviation
RAE: Right atrial enlargement
RBBB: Right bundle branch block
RV: Right ventricle
RVD1: RV diameter basal
RVD2: RV diameter mid
RVD3: RV diameter base to apex
RVFAC: Right ventricle fractional area change
S’ RV: RV pulsed tissue Doppler S wave velocity
RVH: Right ventricular hypertrophy
RVOTP: Right ventricular outflow tract parasternal long axis view
RVOT1: Right ventricular outflow tract proximal
RVOT2: Right ventricular outflow tract distal
SD: Standard deviation
TAPSE: Tricuspid annular plane systolic excursion
TWI: T-wave inversion
WFs: White footballers
